# The perspectives of teachers on barriers to optimum oral health: a qualitative study

**DOI:** 10.1186/s12903-026-08351-1

**Published:** 2026-04-15

**Authors:** Folake Barakat Lawal, Omotayo Francis Fagbule, Adeola Temitope Williams, Taiwo Akeem Lawal, Gbemisola Aderemi Oke

**Affiliations:** 1 Department of Periodontology and Community Dentistry, University of Ibadan and University College Hospital, Ibadan, Oyo State Nigeria; 2 Fellow, Consortium for Advanced Research Training in Africa (CARTA), APHRC, Nairobi, Kenya; 3 Department of Child Oral Health, University of Ibadan and University College Hospital, Ibadan, Oyo State Nigeria; 4 Division of Paediatric Surgery, Department of Surgery, University of Ibadan and University College Hospital, Ibadan, Oyo State Nigeria

**Keywords:** Oral health, Educators, Focus group discussions, Perspectives, Barriers

## Abstract

**Background:**

Poor oral health exists among the Nigerian populace and may be associated with different barriers. Although efforts have been made to institute effective oral health promotion activities among children and adolescents in schools. It remains unclear, from teachers’ perspectives, which barriers to good oral health should be considered when planning such programmes. The aim of this study was to explore the perspectives of school teachers in Ibadan, Nigeria about barriers to good oral health in general.

**Methods:**

This was an exploratory qualitative study conducted among secondary school teachers in Ibadan. Data were collected from a purposive sample of 105 teachers in eight secondary schools in Ibadan through focus group discussions. The discussions were audio-recorded and transcribed verbatim. Thematic analysis was performed.

**Results:**

The ages of the teachers ranged between 35-50 years with about 57.1% of them being females. Individual and community/external barriers were highlighted, and the themes generated included placement of oral health low on priority scale, lack of knowledge about oral health, access to professional dental care services, financial constraint, laziness, and time factor.

**Conclusion:**

In the population studied, insight was gained into the perspectives of teachers about the barriers to optimum oral health.

**Supplementary Information:**

The online version contains supplementary material available at 10.1186/s12903-026-08351-1.

## Background

Teachers play a vital role not only in nation-building but also in promoting the health and well-being of their students and the community at large [1]. This, they achieve by providing accurate and detailed information on preventive health habits, creating a health-promoting environment in the school; screening and referral of students with diseases [2]. In Nigeria, there are about 110 million children and adolescents and approximately 75% of them are enrolled in schools [3, 4]. Given that a significant portion of an individual’s formative years is spent in school, the impact of teachers in instilling healthy habits during this period of life cannot be overemphasized. Meanwhile, this same population has been plagued with several health needs, including oral health needs [5, 6]. Previous studies have documented that adolescents have poor oral hygiene, with a higher prevalence of gingivitis and untreated dental caries [6–9]. This high level of unmet treatment needs suggests the existence of barriers to good oral health.

One of the key implementation strategies of the National Oral Health Policy (2024) is the integration of oral health into school programmes, with the goal that by 2029, at least 50% of schoolchildren will attain optimal oral health through adequate exposure to oral health education and the adoption of healthy oral hygiene practices [10]. 

However, Nigeria continues to face a significant shortage of oral health practitioners [10]. Consequently, the engagement of other key stakeholders, particularly teachers, is essential in supporting the delivery of oral health interventions. Evidence has demonstrated that teachers can effectively contribute to the promotion of oral health within school settings [2, 11]. 

Achieving this target will also require the transformation of schools into health-promoting environments, as recommended by the World Health Organization (WHO). In this context, teachers play a crucial role in fostering supportive environments that encourage and sustain positive oral health behaviours among students [12]. 

Furthermore, the influence of teachers extends beyond the school environment, as students equipped with adequate oral health knowledge can, in turn, promote positive oral health practices among their family members and neighbours [13, 14]. Likewise, teachers are often highly esteemed and regarded as knowledgeable figures within society, placing them in a unique position to influence oral health behaviours within their immediate communities through their own practices and guidance.

Despite the pivotal role of teachers in oral health promotion among students and the community at large, their perspective on the barriers to the oral health of children and adolescents under their care remains underexplored. Therefore, this study aimed to explore the perspectives of secondary schoolteachers in Ibadan, Nigeria, about barriers to optimum oral health. The findings from this study will help in designing appropriate interventions to tackle the barriers to good oral health and in planning effective school oral health promotional programs.

## Methods

This was an exploratory qualitative study conducted among teachers in purposively selected senior secondary schools within Ibadan from January to April, 2023. Ethical approval for the study was obtained from the Oyo State Ethics Review Board (AD 13/479/743). Approval to conduct the study was also obtained from the Oyo State Ministry of Education and from the principal of each of the selected schools. Having explained the purpose and the details of the study, written informed consent was obtained from the purposefully selected teachers who met the eligibility criteria. Only teachers who consented and were available at the time of the discussion were included in the study. Those who were physically indisposed at the time of the study were excluded.

Subsequently, focus groups were constituted with about 8–14 teachers as participants [15, 16]. This approach provides a forum for interactive discussions among the discussants thereby facilitating a clearer understanding of their views on the subject matter [17]. A Focus Group Guide (FGG) was developed based on a review of previously published literatures [18–20]. its face validity was assessed by experts drawn from the University of Ibadan, with postgraduate and clinical fellowship qualifications in the field of Community Dentistry, and by teachers in another school not included in the main study. Thereafter, the FGG was fine-tuned and pretested among five teachers from a school that was also not selected for the main study, to assess its comprehensiveness, and was found to be valid and comprehensive [16]. (Additional file 1)

The Focus group Discussions (FGDs) were conducted in a designated quiet classroom or staff room based on availability. The discussions were moderated in English by one of the authors (FBL) and guided by the pretested FGG. Each session lasted between 25 and 30 min, and was audio-recorded. During each discussion, the moderator paraphrased the information provided by participants and summarized the key findings after each discussion. After the daily FGD session(s), there was a debriefing with a researcher (TAL) [21]. This was done to ensure data completeness and to inform decisions on whether new information needed to be explored in subsequent interviews. The FGDs were continued until no new emerging theme was noted.

### Trustworthiness of data

To ensure rigor and quality of the qualitative data obtained for this study, four criteria; credibility, transferability, dependability, and confirmability were used as described in previous studies [22, 23].

- Credibility: We ensured the credibility of our qualitative study through several rigorous measures. After developing our focus group guide through comprehensive literature reviews, a trained moderator and assistants conducted the discussions. We purposively selected diverse teachers’ participants, focusing on variations in the years of teaching experience We facilitated deep discussion, verified data by summarizing responses for the participants during sessions, and minimized bias by triangulating notes with audio recordings.” During the discussions paraphrasing of the information obtained from the teachers was done at intervals and key findings summarized at the end of each session to ensure that the moderator accurately captures their perspectives and to correct any misinterpretations.
- Transferability: Transferability of our qualitative study was ensured through detailed description of the research setting, the sociodemographic characteristics of the participant and the methodology have been provided. In addition, teachers were selected purposively from different schools and varied based on years of teaching experience.
- Dependability: The details of the various stages of the discussions have been presented to enable replicability.
- Confirmability: The participant pool consisted of secondary school teachers, providing a consistent experiential background. To ensure data triangulation and robustness, the sample was drawn from multiple schools across Ibadan.

### Data analysis

Transcription and validation of audio recordings were done verbatim by a trained research assistant and one of the authors (TAL) compared the accuracy of the transcripts with recordings of the discussions on the audio tapes. Discrepancies noted were corrected after discussions of the research assistant and one of the authors. (FBL) Thereafter, thematic data analysis was done both manually and using NVivo version 12 in six stages which included familiarization with the data, generating initial codes, searching for themes, reviewing themes, defining and naming themes, writing of the report as previously described [24]. 

## Results

A total of 105 teachers participated in eight FGDs. There were 60 (57.1%) females and the mean age of the participants was 40.2 (± 1.2) years. The sociodemographic characteristics of the participants is as depicted on Table 1.

**Table 1:** Sociodemographic characteristics of the study participants

Sociodemographic	FGD 1 *n*	FGD 2 *n*	FGD 3 *n*	FGD 4 *n*	FGD 5 *n*	FGD 6 *n*	FGD 7 *n*	FGD 8 *n*	Total *n* (%)
Gender									
Male	7	6	6	5	6	5	4	6	45 (43.9)
Female	7	7	8	9	8	9	4	8	60 (57.1)
Years of experience									
≤10years	2	1	1	1	1	1	1	2	10 (9.5)
11–20	10	9	9	10	9	8	5	8	68 (64.8)
21–30	2	3	4	3	4	5	2	4	27 (25.7)

Six themes emerged. These are placement of oral health low on priority scale, lack of knowledge about oral health, access to professional dental care services, financial constraint, laziness and time factor. (Table 2)

**Table 2:** Themes and subthemes identified as barriers to optimum oral health by the teachers

Category	Theme	Subtheme
Individual-level barrier	Placement of oral health low on priority scale	Ignorance
		Self-consciousness
	Lack of knowledge about oral health care	Lack of knowledge about correct type of tooth cleaning aids
		Lack of knowledge about correct technique of using tooth cleaning aids
		Lack of proper oral hygiene education
	Financial constraint	Poverty
		Expensive tooth cleaning agents
	Time factor	Time factor
	Laziness	Laziness
Community level / External barrier	Access to professional Dental care	Expensive dental treatment
		Few numbers of dentists
		Inaccessibility to dental care services

### Theme 1. Placement of oral health low on priority scale

The participant mentioned that a barrier to optimum oral health is the placement of oral health low on priority scale by individuals.

#### Sub-theme 1. Ignorance

***“****The number one barrier could be lack of adequate information because most people take oral health for granted”. (Male*,* 40years*,* FGD 4)*

#### Sub- theme 2. Self-consciousness

*………*,* First*,* we don’t put our minds on it [oral care] because we feel that it [oral care] is not always that we should brush and normally we should always realize that it [oral care] is good (Female*,* 42years*,* FGD 1)*.

### Theme 2. Lack of knowledge about oral health

The teachers reported that inadequate knowledge of oral health is a barrier to achieving good oral health.

#### Sub- theme 1. Lack of knowledge about the correct type of tooth cleaning aids

*Another barrier is ignorance when you don’t know when and which product you should use. I mean tooth paste you will usedepend on the problem you have in your mouth or oral care depend on it that you can decide on which product of your toothpaste that can be used. Ignorance will not let you know which one is right and the brush that we use the duration that we should use the brush most of us don’t know some people can use it for years or and instead of may be a month or three months*,* so ignorance is another barrier. (Female*,* 38 years*,* FGD 2)*

*………. I think the challenges are the toothpaste. A lot of toothpaste we have outside. You cannot even identify which one is good or which one is bad. (Female*,* 38 years*,* FGD 3)*

#### Sub- theme 2. Lack of knowledge about correct tooth cleaning technique

*………. an average person*,* when you get to their house*,* they will have toothpaste they will have their brush but for them to know how to use it to brush the correct way is a problem. (Female*,* 38 years*,* FGD 3)*

#### Sub-theme 3. Lack of proper oral hygiene education

*Lack of proper education*,* we are not educated enough on ehn oral hygiene (Female*,* 41years*,* FGD 2)*.

### Theme 3. Financial constraints

Another theme identified was financial constraint and the subthemes are lack of funds, poverty, lack of subsidy of tooth cleaning agents.

#### Sub-theme 1. Lack of funds

*……………. They even recommended that we should brush about two to three times in a day. But if you look at it*,* if you want to follow that step*,* then you must spend more money on purchasing of toothpaste. When you look at the rate with which you are using it*,* you will know that if you follow that trend*,* before you realize*,* before a week or days you know you would have exhausted one so*,* I think it is also a barrier as well. (Female*,* 38years*, *FGD 4)*

*Most need toothbrushes and toothpaste that should improve our oral health due to lack of money. (Male*,* 45years*,* FGD 7)*

*Some people find it difficult to brush their teeth*,* because they do not have enough money to buy toothbrush and tooth paste (Female*,* 42years*,* FGD 5)*.

#### Sub- theme 2. Poverty

*Another barrier could be poverty; some people find it eh… difficult to get reasonable and affordable toothpaste. Although*,* some of them are so cheap but to some people because of the poverty level in the country*,* they are very expensive for some people. for example*,* somebody like me now*,* because of the realities we have in our country*,* there was a time I was using ……. toothpaste*,* but it got to a level that ……toothpaste became “quite expensive”. …………but we have cheaper alternatives that are not as effective as that ……toothpaste*,* you know they are not as effective. So that could be a factor too*,* money*,* lack of information*,* ignorance*,* carelessness and all those things like that. (Male*,* 50years*,* FGD 4)*

*Poverty will not allow one to buy appropriate toothpaste or toothbrush (Female, 40years, FGD 8)*

*………….poverty to buy tooth paste and laziness. (Female, 40years, FGD 6)*

#### Sub-theme 3.Expensive tooth cleaning agents

*Yes*,* I think the first barrier should be finance*,* I think the product of the material being used should make sure the prices shouldn’t be too high for even the poor people and average people can afford it. It shouldn’t be very expensive because these are things everybody need everyday if not twice a day*,* they should make it very available and affordable to everybody. (Female*,* 46years*,* FGD 2)*

### Theme 4.Lack of time

Lack of time to carry out necessary oral health care activities was another theme identified.

*………One because of time factor. We are supposed to brush in the morning and the night last thing in the nights*,* first thing in the morning that’s time barrier. (Female*,* 42years*,* FGD1)*

### Theme 5. Laziness

Another theme identified was laziness, which may make oral care suffer.

*………. the fact that we are lazy*,* laziness also causes it because we may not be able to do the thing at the right time is really a part of laziness that is all*,* I think.(Female*,* 42years*,* FGD 1)*.

### Theme 6. Professional dental care

The teachers opined that professional oral health care services were expensive, and in some cases; inaccessible.

#### Sub-theme 1. Expensive dental treatment

*…………treatment of teeth in the hospital is very very expensive. If they want to clean it. They will charge you*,* so*,* it is very expensive*. *(Male*,* 39years*,* FGD 3)*

#### Sub- theme 2. Short supply of dentists

*Also, I think the dentists. They are not too many (Female, 42years, FGD 3)*

#### Sub- theme 3. Inaccessible dental care services

*There are some hospitals that don’t have dentist department. So*,* those are the major problems that we have. So*,* we cannot locate the dentist easily. (Female*,* 42years*,* FGD 3)*

## Discussion

This study explored the perspectives of secondary school teachers in Ibadan, Nigeria, on barriers to achieving optimal oral health. The findings revealed that most of the barriers were individual-level challenges, including insufficient knowledge, low prioritization of oral health, financial constraints, limited time for twice-daily brushing, and laziness/reluctance towards oral health practices. Additionally, the external barrier identified was poor access to oral care, characterized by a shortage of dental clinics and the high cost of dental care. These findings highlight the multifaceted nature of oral health barriers, emphasizing both behavioural and systemic factors that influence oral health outcomes among the participants.

A lack of knowledge about ideal oral hygiene practices, including the appropriate tooth-cleaning aids and techniques, emerged as a key finding in this study. This aligns with the findings of previous studies in Nigeria [25–27] and in other countries [28–32]. However, in contrast to the findings in this study, some studies reported adequate knowledge among schoolteachers [33, 34]. One such study, conducted in Davangere, India, reported that teachers demonstrated good knowledge of oral health [34]. Notably, this study highlighted that most of the teachers had received oral health training, had topics related to oral health included in the school curriculum, and had taught their students about oral health [34]. While a few other studies [35, 36] have also reported satisfactory knowledge of oral health, a common theme is that teachers may demonstrate good knowledge regarding certain aspects of oral health, such as hygiene, but lack adequate knowledge about the causes of oral diseases. Consequently, these studies still recommend interventions to improve the overall oral health knowledge of teachers [35, 36]. 

Teachers can play crucial roles in advancing health-promoting school initiatives, and their ability to effectively deliver oral health education to students has been recognized for decades [37], and reaffirmed in a recent Nigerian study [11]. Research has shown that teachers are generally willing to undertake this role, provided their capacity is strengthened through appropriate training [34, 36, 38]. Enhancing teachers’ oral health knowledge is essential not only because they serve as educators but also because improved knowledge may positively influence their attitude and personal oral hygiene practices [39]. 

Findings from this study indicated that participants held a negative attitude toward oral health and oral hygiene. This inference is drawn from their expressed low prioritization of oral health, lack of time for twice-daily tooth brushing, and perceived laziness toward oral hygiene practices. Such responses indicate that oral health was not regarded as a significant personal priority. Notably, appropriate tooth brushing takes only about 2 min per session [40], and the inability to allocate time for twice-daily brushing reflects a lack of commitment to basic oral hygiene practices. However, conflicting findings exist regarding teachers’ attitudes toward oral health, which may be attributed to variations in the assessment tools employed across studies [41–43]. 

Financial constraints emerged as a major barrier to accessing dental care, with participants expressing concerns about the high cost of dental treatments, which discouraged routine visits to dental clinics. This finding is consistent with several other studies that, while not conducted among teachers, have shown that affordability remains a significant barrier to dental visits not only in developing countries like Nigeria but also in developed nations [44–48]. Teachers, like many other Nigerians, often lack access to comprehensive health insurance, particularly coverage for basic dental services, further limiting their ability to seek timely oral healthcare [45, 46]. 

We urge the government and relevant stakeholders to intensify efforts in providing adequate and comprehensive dental care insurance for teachers and, indeed, all citizens, to alleviate the burden of out-of-pocket expenses. Additionally, policies and programs aimed at reducing the cost of dental treatment should be prioritized, ensuring that dental facilities are both available and easily accessible to the population [49]. 

The limited availability of dental services was also highlighted as a significant concern. Participants noted that many hospitals lack dedicated dental units, making it difficult for individuals to access professional care when needed. This finding aligns with previous studies that have documented the uneven distribution of dental facilities and professionals in developing and developed countries, where they are concentrated in urban areas while rural communities remain underserved [45, 47, 49–51]. To address this disparity, the Federal Government of Nigeria has committed to providing the necessary support and policy direction to lower tiers of governments and the private sector in their efforts to develop oral health systems that are accessible, equitable, and responsive to the needs of the population [10]. 

Expanding mobile dental clinics and implementing community-based oral health outreach programs could serve as effective short- and medium-term solutions to bridge this gap [52]. 

Additionally, integrating oral health services into the already established and widely distributed primary health clinics offers a sustainable and scalable approach to rapidly improving access to dental care nationwide [49]. 

While this study has identified key barriers to oral health among teachers, it is not without limitations. As a qualitative study involving a limited number of teachers in Ibadan, Nigeria, the findings may not be fully generalizable to the entire country. However, the consistency of these findings with previous research, including quantitative studies conducted in other parts of Nigeria, suggests that the identified barriers are reflective of broader trends in the country’s oral health landscape.

## Conclusion

This study highlights key barriers to optimal oral health among secondary school teachers in Ibadan, Nigeria, including insufficient knowledge, low prioritization, financial constraints, and limited access to dental care. Addressing these challenges requires policy reforms to expand dental insurance, integrate oral health education into school curricula, and improve access through primary healthcare and mobile dental clinics. Given teachers’ role as educators, strengthening their oral health knowledge can have a wider community impact.

## Supplementary Information

Additional file 1.

## Data Availability

The datasets generated and/or analysed during the current study are not publicly available but are available from the corresponding author on reasonable request.
