# Supplementary material for: The perspectives of teachers on barriers to optimum oral health: a qualitative study
Source: BMC Oral Health. 2026 Apr 15;26:1051. doi: 10.1186/s12903-026-08351-1 (PMC13270712; doi:10.1186/s12903-026-08351-1)
Supplement: Supplementary file 1 — Additional file 1. [file 12903_2026_8351_MOESM1_ESM.docx]

**Teachers - Focus group interview guide**

Good afternoon/morning sirs and mas. Thank you for meeting with me today. My name is ……. from ………………………………………. Today I would like to find out what your views/opinions are about the benefits and barriers of optimal oral health. The interview will take about 25 to 45 minutes. I have some specific questions to ask but please feel free to tell me more or provide additional information. Your participation will assist us in evaluating and making improvements to school oral health program. Your answers to my questions will be kept confidential and will not be shared with anyone outside the research team. We will not use your name in any reports or publications.

Do you have any questions?

I would like to record the interview to ensure we capture all the details. Is it alright if I record? Can we proceed PLEASE?

Thank you sirs and mas.

1. How did you feel when you received our invitation that we will be coming to your school?
2. When you received the message of invitation that we will be coming to your school, what do you think or what came to your mind?
3. Does taking care of the health of the teeth and mouth of any benefit?
4. If yes, why?
5. If no , why?
6. What are the barriers to good health of the mouth and teeth that is the barriers that will not let us take care of our teeth and mouth regularly?
